# Synthesis of biocompatible Konjac glucomannan stabilized silver nanoparticles, with *Asystasia gangetica* phenolic extract for colorimetric detection of mercury (II) ion

**DOI:** 10.1038/s41598-022-13384-x

**Published:** 2022-06-02

**Authors:** Titilope John Jayeoye, Fredrick Nwude Eze, Opeyemi Joshua Olatunji, Andrew Aondoaver Tyopine

**Affiliations:** 1 Department of Chemistry, Faculty of Physical Sciences, Alex-Ekwueme Federal University, Ndufu-Alike Ikwo, P.M.B. 1010, Abakaliki, Ebonyi State Nigeria; 2 Faculty of Pharmaceutical Sciences, Prince of Songkhla University, Hat Yai, 90112 Songkhla Thailand; 3 Drug Delivery System Excellence Center, Prince of Songkhla University, Hat Yai, 90112 Songkhla Thailand; 4 Traditional Thai Medical Research and Innovation Center, Faculty of Traditional Thai Medicine, Prince of Songkhla University, Hat Yai, 90110 Thailand

**Keywords:** Chemistry, Nanoscience and technology

## Abstract

Herein, the synthesis of a biocompatible silver nanoparticles (AgNPs), for colorimetric detection of toxic mercury (II) ion (Hg^2+^), is reported. Phenolic-rich fraction of *Asystasia gangetica* leaf was extracted and used as a reductant of silver salt, all within the hydrophilic konjac glucomannan (KgM) solution as stabilizer, at room temperature (RT). The bioactive components of *Asystasia gangetica* phenolic extract (AGPE), as elucidated with a (UHPLC-MS-QTOF-MS), revealed plethora of phenolic compounds, which can facilitate the reduction of silver salt at ambient conditions. Sparkling yellow colloidal solution of KgM-AgNPs was realized within 1 h, at RT, having a UV–vis maximum at 420 nm. KgM-AgNPs was characterized using UV–vis, Raman and (FTIR), TEM, SEM, EDS, XRD, TGA/DTG. TEM and FESEM images showed that KgM-AgNPs were spherical, with particle size distribution around 10–15 nm from TEM. The KgM-AgNPs biocompatibility was investigated on mouse L929 fibrobroblast and rat erythrocytes, without any harmful damages on the tested cells. In aqueous environment, KgM-AgNPs demonstrated good detection capacity toward Hg^2+^, in a Hg^2+^ concentration dependent fashion, within 3 min. Absorbance ratios (A_360_/A_408_) was linear with Hg^2+^ concentrations from 0.010–10.0 to 10.0–60.0 µM, with an estimated (LOD) of 3.25 nM. The probe was applied in lake water sample, with satisfactory accuracy.

## Introduction

One of the major revolutions in chemistry over the past few decades is in the application of metal nanoparticles (MNPs) in various analytical applications. As a notable nanomaterial, with sizes in the nanometers (nm) range, they possess unrivalled properties in comparison with common available fluorophores. These properties include, localized surface plasmon resonance (LSPR) phenomenon, high extinction coefficients, catalytic ability, unique color display, tunable surface morphologies with common recognition materials^1^. The mentioned properties are all heavily correlated with their particle size, shape, charge, the dielectric of the medium in which they are embedded, temperature, their surface coatings, among others^2,3^. In view of the foregoing, the application of MNPs particularly of silver, gold, copper have consistently been attracting attentions in the scientific community. For instance, surface functionalization/modification and stabilizers of silver nanoparticles play critical roles in determining detection sensitivity and selectivity to wide ranging analytes^4,5^^,^^6^. This step also plays important role in modulating nanoparticles biocompatibility^7,8^. It is also a common practice to tune nanoparticles surfaces with ligands having specific affinity with the analyte of interest. As such, the injection of the sensing analyte could induce significant changes to the optical properties of the modified nanoparticles.

Pollution of major environmental matrices (air, water and soil) is an intractable challenge to the ecosystem in view of the humongous number of wastes generated from human activities. Regrettably, some of such wastes with toxic profiles are not properly managed to safeguard the environment. Heavy metal pollution is one of such, and it remain so, partly owing to the high-level resource’s exploitation at industrial scale in a bid to provide fundamental needs. From the perspective of environmental safety, heavy metals (HMs) are defined as metals with inherent capacity to induce eco-physiological damages arising from their high toxicity^9^. In this regard, metals such as mercury (Hg), lead (Pb), silver (Ag), cadmium (Cd) and chromium (Cr) are mostly implicated. Among these HMs, special interest is devoted to Hg- because of its non-biodegradability and adverse bio-accumulative potentials. In fact, the Hg contamination cycle is disturbing. For instance, Hg contamination of water bodies, can lead to hyper accumulation of the metal in fish and other aquatic animals which when consumed by humans, could elicit deleterious health consequences. The popular Minamata disaster in Kumamoto prefecture, Japan, where methyl mercury (CH_3_Hg) contamination of water, arising from wastewater from an adjoining chemical company, led to deaths of pets and severe health challenges in humans^10^. The foregoing, illustrated the importance of Hg monitoring in the environment for overall well-being of humans and other animals.

Hg contamination takes two major forms; organic and inorganic. The organic form includes: Hg^2+^, Hg^+^ and Hg^0^, while alkyl mercury, with CH_3_Hg a prominent form, account for the inorganic form of Hg^11^. Among the list, CH_3_Hg and Hg^2+^ are the most toxic^12^, owing to their effect on the human organs such as: the kidney, liver, brain and the entire central nervous systems (CNS), arising from their high binding capacity to thiols and enzymes, thereby obstructing enzymes optimum functions^13^. It is germane to emphasize that Hg^2^^+^ is the most abundant form of Hg in water^14^, as a result, the maximum allowable concentration of Hg^2+^ in drinking water is fixed at 10 nM by the United States Environmental Protection Agency (USEPA), while the world health organization (WHO), set same limit as 30 nM. These low regulatory limits, require careful design of analytical methods with high sensitivities to be applied for Hg^2+^ quantification and monitoring in real samples. Reported detection strategies for Hg^2+^ include atomic absorption spectroscopy (AAS)^15^, inductively coupled plasma-atomic emission spectroscopy (ICP-AES)^16^, colorimetric detection using gold and silver nanoparticles^17–20^, fluorescence-based assays^21,22^ and electrochemical sensors using modified electrodes^23,24^. These methods have all been successfully used for reliable detection of Hg^2+^ in different matrices, however, some inherent challenges can be spotted. Methods using analytical equipment such as AAS and ICP methods require rigorous sample preparations and thus may pose difficulty, for applications involving on-site activities. Moreover, the limit of detection reported in some of the works are high, thus, new methods are regularly investigated by researchers, to combine simplicity, sensitivity and environmental friendliness.

Biopolymers are naturally occurring macromolecules with sources traceable to plants, animals or micro-organisms^25^. There is indeed huge collection of materials under this category, with examples such as: alginate, carboxymethyl cellulose, chitosan, fucoidan, carrageenan, pullulan, konjac glucomannan etc., all well exploited for diverse applications. Konjac glucomannan (KgM), derived generally from the tuber of (*Amorphophallus konjac*), is one of the common available biopolymers, which have been well popularized for its perfect aqueous dispersity and its gel/film-forming property^26^. From its structural perspective, KgM consist of 1,4-β-D-mannose and a β-D-glucose unit, with a ratio of 1.6 to 1, in addition to some acetyl groups^27^. These properties, would imbue aqueous solution of KgM with appropriate features to stabilize colloidal silver nanoparticles, while also contributing to its biocompatible properties.

*Asytasia gengetica* or Chinese Violet, is a common weed, of the Acanthaceae family, predominant in tropical African and Asian continents^28^. It is a rapidly growing plant, even on soils with low fertility and in shades. In fact, it is regarded as a notorious weed, competing for soil nutrients (nitrogen and phosphorus), with crops, thus reducing productivity. In Australia, *A. gangetica* is on the list of Alert Environmental weeds^29^, owing to its ability to grow and spread, even under unfavorable conditions. Unfortunately, only few research works have been devoted to the elucidation of the active metabolites present in this plant.

Silver nanoparticles (AgNPs) synthesis and applications have taken an enviable position amongst various nanomaterials available. This may be due to the ability of AgNPs to be used as an antibacterial agent (depending on the material deployed for its synthesis)^30–32^, and its localized surface plasmon resonance property (LSPR), which can be tuned for the sensing/detection of various analytes^33–35^. The ability of AgNPs to serve the aforementioned roles is highly dependent on the precursor materials used for its fabrication, the synthesis conditions, storage conditions etc. Two common synthesis strategies are quite popular nowadays, viz: chemical and biological/green synthesis. The use of chemical reducing agents are being criticized from the prism of environmental safety, while green synthesis remains the cornerstone of nanofabrication in the present century.

In this contribution, we have conceptualized the use of phenolic extract from a notorious garden weed, *A. gangetica*, for the reduction of silver salt inside KgM biopolymer matrix, to generate biocompatible KgM-AgNPs, at ambient condition. Summarily, AGPE served as reductant of silver salt, while KgM was applied as a shape directing specie/stabilizer of the realized AgNPs. The synthesis was carried out at room temperature, without the use of any harmful chemical reductants, the first of KgM application for AgNPs synthesis at RT/ambient condition. The fabricated KgM-AgNPs was applied as colorimetric probe towards the detection of toxic Hg^2+^ with satisfactory analytical performances.

## Materials and methods

### Materials

Konjac glucomanan (KgM), with 95.8% purity, was from TCS-Mart, Thailand. (0.25% w/v KgM, 23.0 $^\circ$C had viscosity of 218.0 cP, Mwt was of 250 kDa). AgNO_3_ (analytical grade), Cr (NO_3_)_3_, HgCl_2_ were from Sigma Aldrich, other metal salts include KNO_3_, NaNO_3_, ZnSO_4_.7H_2_0, Ca (NO_3_)_2_.4H_2_O, CoCl_2_.6H_2_O, CdCl_2_.2.5H_2_O, CuSO_4_.5H_2_O and Al (NO_3_)_2_.9H_2_O from APS Ajax Finechem, MnCl_2_.4H_2_O from QReC, Pb (NO_3_)_2_ was from Spectrum Chemical, Fe_2_SO_4_.7H_2_O from Merck, while FeCl_3_.6H_2_O, MgCl_2_.6H_2_O and NiCl_2_.8H_2_O were from LOBA CHEMIE. The listed chemicals were used for the selectivity and interference studies. All reagents were prepared using milli-Q water from Millipore water purifier system.

### Plant extracts preparation

Fresh aerial part of *A. gangetica* was collected on campus and quickly processed as earlier described^36^. Succinctly, the plant sample was oven-dried at 50 °C to constant weight. The plant material was then pulverized using an electric grinder to fine powder. *A. gangetica* powder was subsequently extracted with 70% ethanol at a solid to solvent ratio of 1:10 for 2 h using an overhead stirrer at room temperature. The mixture was filtered using Whatman No.4 filter paper. The extraction was repeated on the marc, and the filtrate was combined. The combined filtrate was further filtered under gravity using Whatman No.1 filter paper and concentrated using rotary evaporator. The filtrate was then partitioned by cold fractionation to collect the hydropilic fraction. This fraction was lyophilized to obtained *A. gangetica* phenolic-rich fraction.

### UPLC-ESI-QTOF MS characterization of A. gangetica extract

The detailed phytochemical profile was determined qualitatively by UPLC-DAD-ESI-QTOF-MS/MS analysis to obain an overview of the individual bioactives presnt in the extract. Briefly, appropriate amout of the extract was carefully weighed and solubilized in 70% methanol. This solution was mixed by vortexing for 5 min. Thereafter, the solution was centrifuged at 7168 xg for 5 min. The supernatant was collected and syringe filtered through a nylon membrane (0.2 µm). The clear solution was then immediately subjected to LC–MS analysis^37^.

### Total phenolic content

The total phenolic content of A. gangetica was determined as detailed in previous work^38^. Briefly, 100 µL of aqueous solution of A. gangetica extract (the extract was soluble in water) or gallic acid (standard) was added into 2 mL Eppendorf tubes. Then 200 µL of 10% Folin-Ciocalteu reagent was added to the solution and mixed. After 5 min, 800 µL of freshly prepared sodium carbonate solution (700 mM) was added to the mixture and mixed by vortexing. The sample and standard solutions were then incubated in the dark at room temperure for 2 h. Subsequently, absorance of the solutions were read at 765 nm. Gallic acid yielded a standard curve with linearity between 0.2 to 0.01 µg (R^2^ = 0.9985). The total phenolic content of A. gangetica extract was extrapolated from the gallic acid standard curve.

### Synthesis of KgM-AgNPs

For the synthesis of KgM-AgNPs, an 0.25% w/v of KgM was prepared by dissolving 0.25 g of KgM in 100 mL water under vigorous stirring at RT. After 20 min, the solution was heated to 60 $^\circ$C and was maintained for 1 h. Afterwards, the viscous solution was cooled to RT before use for KgM-AgNPs synthesis. Aqueous solution of AGPE powder used as reductant, was prepared in water/ethanol mixture (3:1). Into a 200 mL beaker, wrapped with aluminum foil, 93 mL of KgM solution (0.1%), was added under stirring, then 2 mL of AgNO_3_ of different concentrations, were added, for synthesis optimization. The mixture was blended for 5 min, after which, 5 mL of AGPE (whose pH has been pre-adjusted using 0.1 M NaOH solution), was injected. The final solution was maintained under stirring at RT for further 60 min. The sparkling yellowish colloidal solution of KgM-AgNPs was stored at 4 °C before use. The colloidal solution was diluted before the UV–vis spectra were acquired. For biocompatibility test, the concentration of Ag in the prepared material was estimated with ICP-OES, then serial dilutions were prepared and used for the assay. For characterizations, KgM-AgNPs was lyophilized and the obtained film was used for material characterization.

### Biocompatibility test

*Hemolytic assay.* The potential adverse effect of AGPE-AgNP on red blood cells was evaluated in vitro as previously described^38^. The nanoparticles, extract or KgM, 0.10% (100 µL) was incubated with dilute samples of freshly collected erythrocytes (400 µL) for 60 min at 37 °C. Thereafter, the solutions were centrifuged at 112 xg for 5 min. The supernatants were collected and the OD taken at 540 nm. As positive control 100 µL of distilled water (DW) was used instead of the sample, while phoshate buffer saline (PBS), pH 7.4 was used as negative control. The extent of erythrocyte hemolysis was represented in percentage.

*Cytotoxicity assay.* The potential cytoxicity of the nanoparticles was determined by evaluating its effect on the viability on mouse L929 fibrobroblast. The cells were seeded (1.5 × 10^4^ cells/ well) in 96-well plates and incubated for 24 h in a 5% CO_2_ humidified incubator. Subsequently 100 µL solution of nanoparticles, extract or KgM was added to the wells. The plates were further incubated for 24 h and the viability of the cells were determined by MTT assay according to the manufacturer’s protocol (Sigma-Aldrich Cell Proliferation Kit I).

### Instrumental characterizations

All absorption spectra were acquired on a SPECTROstar Nano/BMG LABTECH UV–vis spectrophotometer, with a 1 cm pathlength glass cuvette and distilled water as the solvent. Transmission electron microscope (TEM), images were observed using a JOEL, JEM 2010 from Japan. About 5 µL of the nanoparticles was dropped on TEM copper grid and was allowed to dry in a desiccation 48 h before images observation. At least, three images were captured at each magnification. Size distribution histogram was plotted after measuring about hundred particles using an Image J software. FEI Apreo (Czech Republic), Field emission scanning microscopy (FESEM) was used for SEM images observation. The sample film was dropped on SEM aluminum stub, while three replicate images were equally acquired at different magnification. The equipment is attached to an energy dispersive X-ray spectroscopy (EDX) facility from (X-Max 80, Oxford instruments, UK). Raman Spectra of samples were acquired on a Raman Microscope Spectrometer, RAMANforce, Nanophoton, Japan. X-ray diffraction (XRD) was obtained using an Empyrean XRD diffractometer, from 2 theta (degree), range 5–80$^\circ$, applying a step size (2θ) of 0.026$^\circ$, time/step value of 70.125 s, scan speed of 70.2 s, with Cu Kα radiation value of 0.154 nm. Brookhaven Nano Brook ZetaPALS potential analyzer (USA) was used for the hydrodynamic diameter measurement of zeta potential acquisition. The as synthesized KgM-AgNPs was diluted five-fold using distilled water. The mixture was poured into DLS plastic cuvette of 5 mL capacity and was then inserted into the sample chamber. The machine was set for sample run, n = 10 at 25 °C and each sample was run in triplicates. Further, Attenuated Total Reflectance-Fourier Transform Infrared Spectrometer (ATR-FTIR), Vertex70, Bruker, Germany, was used for functional group elucidation, acquired within 400–4000 cm^−1^ for samples. The thermal stability of the test samples was acquired suing a thermogravimetric analyzer, TGA8000, Perkin Elmer USA. Certain mass of the samples was subjected to heating from 50 to 1000 $^\circ$C at the rate of 10 $^\circ$C/min in nitrogen.

### Analytical detection of Hg^2+^ in solution

Hg^2+^ detection using KgM-AgNPs was realized as follows. Into a 2 mL colorimetric tube, 600 µL of KgM-AgNPs colloidal solution was pipetted, 1 mL of phosphate buffer solution (PBS) 50 mM, pH 6.0 was added, 200 µL of Hg^2+^ of different concentrations were injected (final concentration 0.0 to 60.0 µM), followed by the addition of 200 µL of 0.25 M NaCl solution (final concentration 0.025 M). The mixture was vortexed, while the photo images and absorption spectra were collected, after 3 min incubation at RT, using a Samsung phone A50 and a UV–vis spectrophotometer respectively.

## Results and discussion

### Chemical characterization of AGPE

The specific type or group of chemical compounds in a plant extract prepared for green synthesis of noble metal nanoparticles plays an important role in determining the actual synthesis efficiency^39^. Plant extracts contain a diversity of bioactives, and the most abundant amongst these is often a function of the particular plant as well as the source and condition under which it was cultivated. There is growing evidence that phenolic compounds are highly efficacious as green reductants. Data from the Folin-Ciocalteu assay revealed that *A. gangetica* extract is rich in phenolic constituents with a total phenolic content of 135.46 ± 1.01 mgGAE/g AGPE d.w.

With regards to the identities of the individual bio actives present in AGPE, ultra-high-performance liquid chromatography coupled to tandem mass spectrometry (UPLC-ESI-QTOF-MS/MS) analysis was performed for their elucidation. The mass spectrometry was performed in the negative ion mode. A total of 17 compounds were identified in AGPE (Table S1). These compounds were found to belong to various sub-groups of phenolics such as flavonoids, lignans, and phenolic apioglycosides. Compound #1, yielded a parent ion with an accurate mass of 478.1695 and m/z ratio at 523.1677, which corresponded with [M + HCOO-]-, i.e., the formate adduct of kelampayoside A. The identity of phenolic apioglucoside was established based on the obtained MS data and literature report^40^ and has been previously described as a strong radical scavenging agent. Other phenolic glycosides observed in AGPE include bufotenine O-glucoside^41^, verbasoside or decaffeoylverbascoside (a phenylpropanoid glycoside) with major peak at m/z 461, corresponding to the deprotonated pseudomolecular ion [M-H]-^42^; trans-p-Coumaric acid 4-glucoside, Quercetin 3-[p-coumaroyl-(- > 6)-glucosyl-(1- > 2)-glucosyl-(1- > 2)-glucoside] (a flavonoid-o-3-glycoside); Glucoliquiritin apioside (a flavonoid-7-o-glycoside) and the lignan glycoside, 8-Acetoxypinoresinol 4-glucoside. Beside the phenolics, other compounds such as the diterpene glycoside, 19-Hydroxycinnzeylanol 19-glucoside as well as the monoterpenoid iridoid-o-glycoside, caryoptosidic acid^43^ and fatty acyl glycoside, 1-Octen-3-yl primeveroside were also present in AGPE. The presence of these compounds, especially the phenolics, provided a strong indication of the metal nanoparticle biosynthetic capacity of A. gangetica extract.

### AGPE based synthesis of KgM-AgNPs

**Figure 1:**
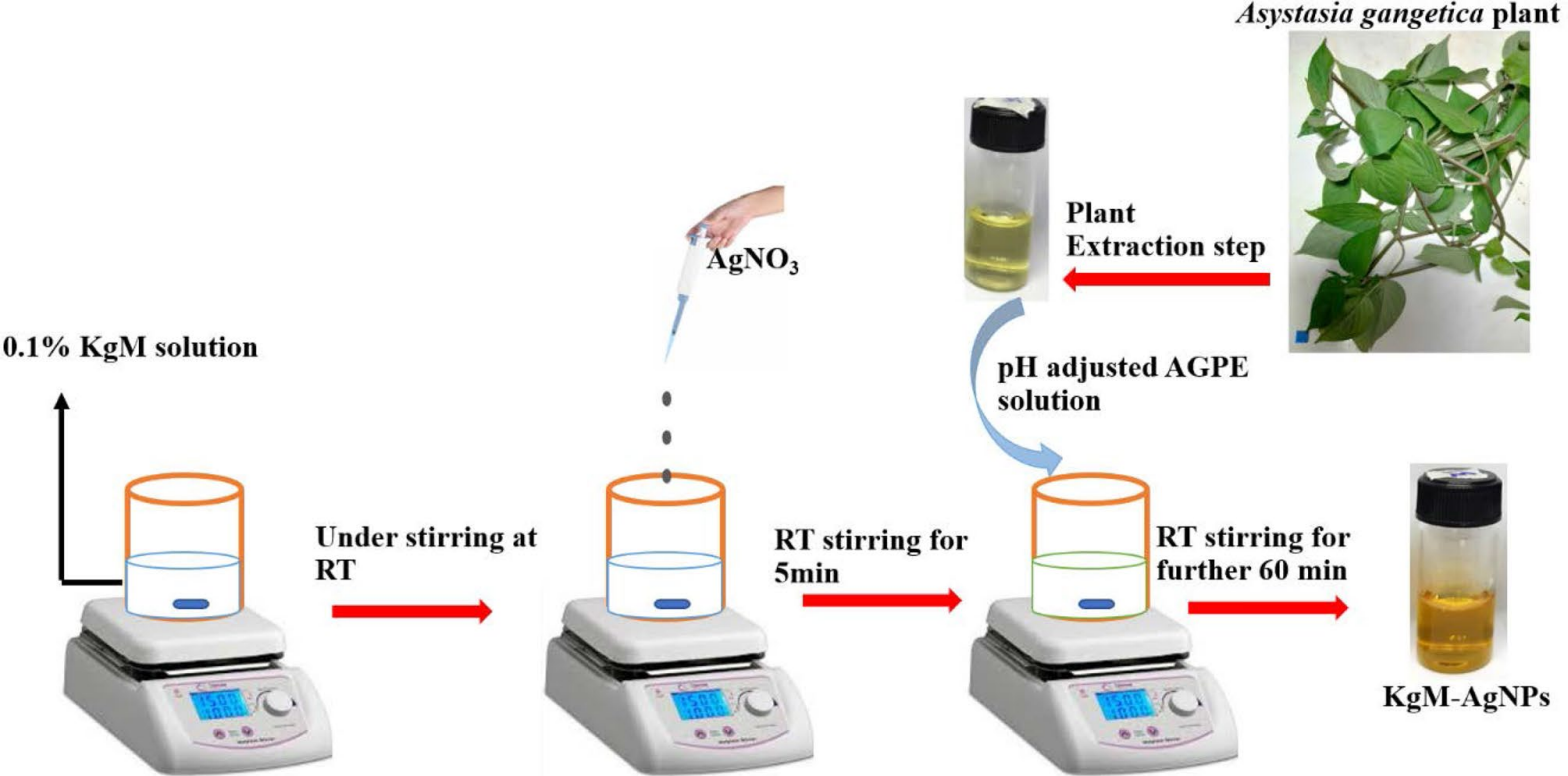
Schematic illustration of the synthesis strategy of KgM-AgNPs at room temperature (RT).

Under one-pot synthesis, using AGPE as the reductant of AgNO_3_, while applying KgM as the stabilizer, KgM-AgNPs could be realized under 1 h, at ambient condition (RT and stirring), Fig. 1. In fact, the addition of pH adjusted AGPE into a mixture of KgM and AgNO_3_, resulted in instantaneous yellow color, which is attributed to the rich bio actives present in AGPE, while the nanoparticles nucleation and growth process, could be completed in 1 h. AGPE bioactive constituents can facilitate the reduction of silver salt, without the use of external agents, unlike what has been reported. Jian et al., reported the synthesis of KgM stabilized AgNPs, through photocatalytic ultraviolet irradiation^44^, while Chen et al. reported a KgM-AgNPs realized through heating of KgM and AgNO_3_ at 60 °C for 30 mins^45^. These works showed that the reduction efficacy of KgM is limited at ambient conditions, which is equally peculiar to other biopolymers.

**Figure 2:**
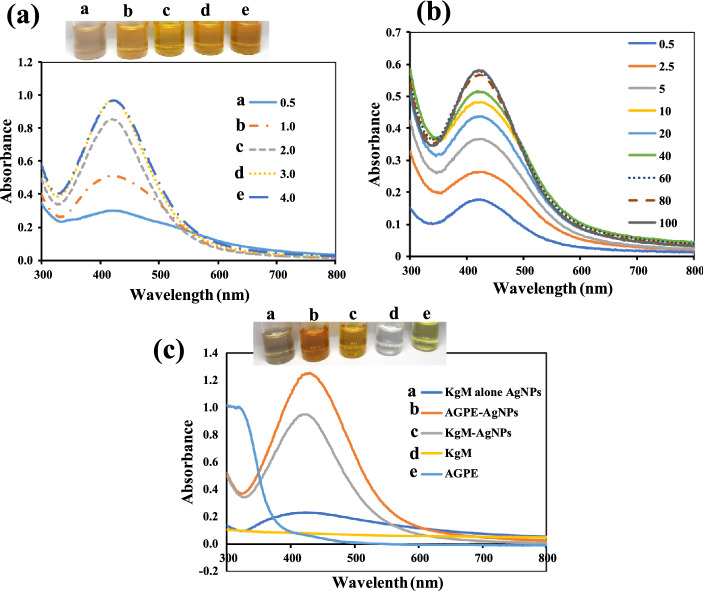
UV–vis absorption spectra of (**a**) Colloidal KgM-AgNPs realized using different concentrations of AgNO_3,_ a 0.5 b1.0 c 2.0 d 3.0 and e 4.0 mM, while inset shows the photo images, (**b**). UV–vis absorption spectra of 2.0 mM AgN0_3_, synthesis kinetics (0.5 to 100 min), selected as optimal condition and (**c**). UV–vis absorption spectra of materials and silver nanoparticles realized using varying materials, a. AgNPs using KgM alone b. AgNPs using AGPE alone (**c**). AGPE based synthesis of KgM-AgNPs d. KgM biopolymer and e. AGPE, while inset shows the photographic images of the different materials.

In this work, we found the concentrations of AgNO_3_ in the final reaction pot as key to the realization of a stable colloidal solution with the best properties and was thus optimized. As shown in Fig. 2a, as the concentration of AgNO_3_ increases, the absorption spectra of the solution increased, which is attributed to the formation of more AgNPs. The photo image of the solution equally changed from grey to sparkling yellow (Fig. 2a inset). The absorption maximum realized, under different concentrations of AgNO_3_, is depicted as; 0.5 (424 nm), 1.0 (424 nm), 2.0 (420 nm), 3.0 (421 nm) and 4.0 mM (422 nm). In order to obtain more information on the dispersity and stability of the realized AgNPs, the absorbance ratio (A_420_/A_650_) plotted against the different concentrations of AgNO_3_ was obtained (Fig. S1). Accordingly, AgNPs synthesized with final AgNO_3_ concentrations yielded (A_420_/A_650_) ratios; 0.5 (3.670), 1.0 (11.568), 2.0 (19.386), 3.0 (17.053) and 4.0 mM (14.893), respectively. From the foregoing, using AgNPs with the least absorption maximum and the highest (A_420_/A_650_) value, silver nanoparticles synthesized with final AgNO_3_ concentration of 2.0 mM, was selected as the optimal synthesis condition. It must be pointed out, that the concentration of KgM was fixed at 0.10% in all synthesis condition.

To study the synthesis kinetics under the optimal condition, the absorption spectra were collected from 0.5 to 100 min, as shown in Fig. 2b. As revealed, the absorption intensity increased over time, then plateaued at 60 min. The plot of A_420_ nm against time is shown in Fig. S2, which revealed intensity saturation at 60 min.

Furthermore, the roles of the reacting species, on the RT synthesis, were investigated and shown in Fig. 2c. As revealed, AgNPs synthesis using the biopolymer alone generated cloudy brown solution (Fig. 2c (a inset)). It is worth pointing out, that the solution was not diluted, in comparison with that acquired using AGPE alone and KgM-AgNPs. AgNPs synthesis using AGPE alone in the absence of the biopolymer showed, maximum absorption spectrum at 429 nm, with a darker yellowish color (Fig. 2c (b inset)). Synthesis generated with AGPE and the biopolymer KgM, showed a blue shift, with maximum absorption spectra at 420 nm (Fig. 2c) though with decreased intensity. This is attributed to the dense polymer layer on the nanoparticle’s surfaces. This observation has been reported^46^. The blue shift also validated the role of KgM as not only providing further stabilization for the synthesized KgM-AgNPs, but also serving as shape-directing species, since a blue shift may be correlated with smaller particles diameter. The photo image obtained under this condition (Fig. 2c (c inset)), showed a fainter yellowish colloidal dispersion. The absorption spectra and photo images of KgM (Fig. 2c (d inset)) and AGPE (Fig. 2c (e inset)), do not present any notable characteristic maximum absorption spectra within 400–450 nm, while the pale-yellow color of AGPE can be easily differentiated from sample nanoparticles.

The stability of the synthesized KgM-AgNPs was monitored for twenty-six weeks (Fig. S3). It did show, that there is no development of any peaks at a longer wavelength, peculiar to aggregated nanoparticles, hence, the fabricated KgM-AgNPs is highly stable. This may be due to the synergistic effects of AGPE and KgM, which can modulate the surface energy of the nanoparticles by preventing facile particles agglomeration.

From the foregoing, it is safe to propose that the addition of pH adjusted AGPE solution to a mixture of AgNO_3_ and KgM, the deprotonated multitudinous phenolics in AGPE will release electrons for Ag^+^ reduction to Ag^0^, all inside KgM matrix. Further, the KgM matrix, can enhance/facilitate the nucleation and growth process and thus modulate synthesis process. With this, AGPE would act as a facile reducing agent, while KgM served as stabilizer, towards KgM-AgNPs synthesis.

### Characterization of silver nanoparticles

**Figure 3:**
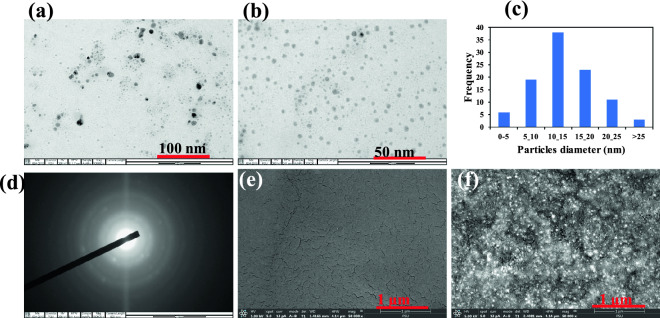
TEM images of KgM-AgNPs at (**a**) 100 nm and (**b**) 50 nm (**c**) Particles size distribution obtained from ImageJ software (ImageJ-1.38e: https://imagej.nih.gov/ij), (**d**) Selected area electron diffraction (SAED), FESEM images of (**e**) KgM and (**f**) KgM-AgNPs.

To gain deep insight into the microstructure of materials and the nanoparticles fabricated, various instrumental characterizations were applied. The TEM images of KgM-AgNPs obtained from the optimal synthesis conditions is shown in Fig. 3a,b. As can be seen, the particles are perfectly mono-dispersed with spherical morphologies. The average particles size obtained from TEM, using Image J software, revealed the particles are distributed between 10–15 nm (Fig. 3c). The hydrodynamic diameter from DLS, was found to be 60.2 ± 1.5 nm (Fig. S4), while the zeta potential value was −28.8 ± 2.1 mV. This shows that the particles are stable, with negative charges, which may be from the carboxyl group of KgM polymeric spheres/shell. The effect of pH on the aqueous stability of KgM-AgNPs, through Zeta potential profiling is shown in (Fig. S5). As shown, the zeta potential values increase as the solution pH increases from 2 to 4 and stabilized afterwards till 12. This shows KgM-AgNPs is stable over a wide pH range, which may be attributed to the deprotonated carboxyl group of KgM on the nanoparticle’s surfaces.

The selected area electron diffraction image (SAED) showed various concentric circles, which is attributed to the crystalline diffraction lines of AgNPs. To further obtain information on KgMP-AgNPs, FESEM images of KgM and KgM-AgNPs were acquired (Fig. 3e,f). As shown, KgM film showed smooth morphology (Fig. 3e), while KgM-AgNPs displayed rough morphology, with plethora of interspersed AgNPs (Fig. 3f). These show that the fabricated AgNPs are firmly protected by the polymeric layers provided by KgM biopolymer.

**Figure 4:**
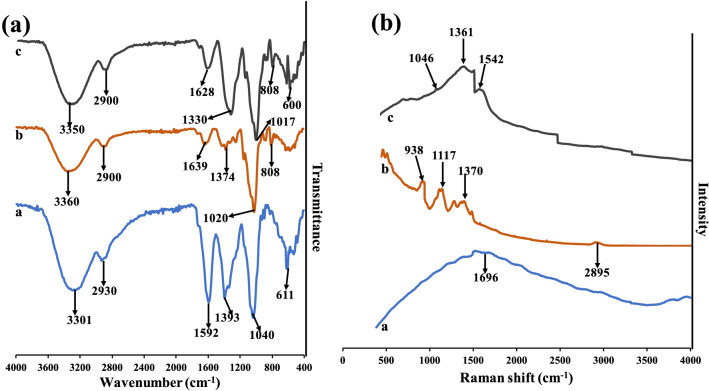
(**a**) FTIR spectra and (**b**) Raman spectra of (**a**) AGPE (**b**) KgM c KgM-AgNPs.

Further, functional groups interplay between the synthesis materials (AGPE, KgM and KgM-AgNPs), were revealed using FTIR and Raman spectroscopy. The FTIR spectra of AGPE (Fig. 4a a), shows major peaks at 3301, 2930,1592,1393,1041 and 611 cm^−1^. These peaks are assigned to the C–OH stretching of OH groups from phenolic rich compounds, C–H stretching of aliphatic groups, C=C or C=O groups of amide for 1592 cm^−1^^47^, peaks at 1393 is attributed to C–C stretching of ring structure in aromatic compounds, while peaks at 1041 and 611 are assigned to C–O–C and C–OH stretching of secondary alcohols and C–Cl respectively^48^. Major peaks in KgM (Fig. 4a b) are, 3360, 2900, 1639, 1370, 1020 and 808, which are assigned to the OH groups on KgM, C–H stretching of methyl or methylene group, C=O at1639 cm^−1^, C–O stretching vibrations, while peaks at 1020 and 808 cm^−1^, are characteristic peaks of the glycosidic linkages of the polymeric structure of KgM^44^. The peaks in KgM-AgNPs (Fig. 4a c), at 3350, 2900, 1628, 1330, 1017, 808 and 600 cm^−1^ are reflective of characteristic peaks identified in AGPE and KgM. This validated the synergistic combination of AGPE and KgM, through hydrogen bonding interaction, is implicated in KgM-AgNPs fabrication. This observation was noted by Tian et al.^49^.

The Raman spectra is displayed (Fig. 4b). Major peak at 1696 cm^−1^ was identified in AGPE (Fig. 4b a), which is attributed to the O–O vibration, while peaks at 938, 1117, 1370 and 2895 cm^−1^ are conspicuous in KgM (Fig. 4b b). These peaks (938 and 1117) are assigned to the C–O–C vibrational bands of KgM glycosidic linkages, while bands at 1370 is from the vibrational methyl group in acetyl moiety and the peak at 2895 is from the C–H stretching modes^50^. Figure 4b c), shows the Raman spectra of KgM-AgNPs, with peaks at 1046, 1361 and 1542 cm^−1^, which are similar to the peaks identified in KgM and AGPE. This similar trend was observed in FTIR results discussed supra.

**Figure 5:**
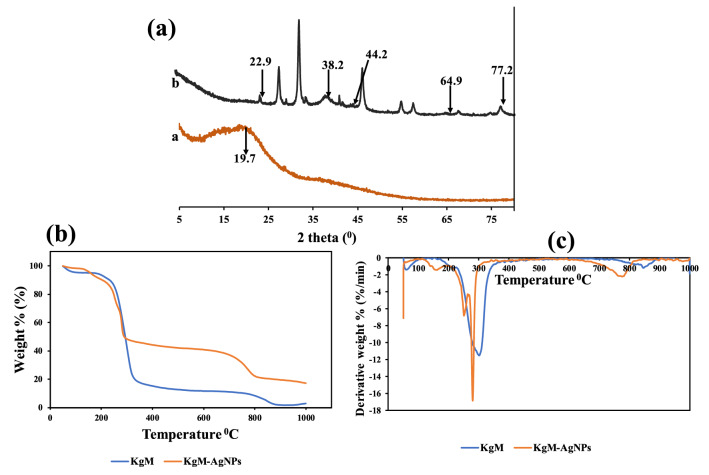
(**a**) XRD of a KgM b KgM-AgNPs (**b**) TGA and (**c**) DTGA of KgM and KgM-AgNPs.

The XRD patterns of KgM and KgM-AgNPs are displayed in Fig. 5a. As revealed, major diffraction peak of KgM at 19.7$^\circ$ (Fig. 5a a), which is atypical of the amorphous structure of KgM, while peaks at 22.9, 38.2, 44.2, 64.9 and 77.2$^\circ$ in KgM-AgNPs, are assigned to KgM and (111), (200), (220) and (311) of face centered-cubic diffraction planes of AgNPs^44^. The Scherer equation was used to calculate the crystallite size of the material using the (111) plane of silver. The equation used, D = kλ**/**βCosθ, yielded a crystallite size estimation of 17.2 nm which is close to the average estimated size from TEM.

The elemental compositions using energy dispersive x-ray spectroscopy (EDS), of the materials KgM and KgM-AgNPs are displayed in Fig. S6. Accordingly, elements C and O with 71.2 and 28.8% are present in KgM (Fig. S6a), while elements Ag, C and O with 77.7, 15.2 and 7.1% respectively are identified in KgM-AgNPs (Fig. S6b). Moreover, sharp Ag peaks at about 3.0 kev is observed in KgM-AgNPs (Fig. S6b). This validated the successful synthesis of AgNPs.

The elemental mapping of KgM and KgM-AgNPs are displayed in Fig. S7. The results confirmed elements C and O for KgM (Fig. S7a) and Ag, C and O for KgM-AgNPs (Fig. S7b), in accordance with the EDS result above.

The thermal stability of materials may convey information on the roles of the constituting materials, in the overall final products. Figure 5b,c, show the TGA and DTGA of KgM and KgM-AgNPs. As can be observed, three degradation steps can be identified in the thermal degradation profile of KgM and KgM-AgNPs (Fig. 5b). These stages are summarized in Table S2. As shown, the onset degradation temperature of KgM was observed at 51.4 °C. The breakdown are as follows. Stage I (51.4–259.8 °C), stage II (259.8–386.2 °C) and stage III (386.2–999.3 °C). The total loss is about 97.97%, with ash content of 2.03%. However, three stages in KgM-AgNPs are: stage I (51.5–237.7 °C), stage II (237.7–341.5 °C) and stage III (341.5–999.3 °C). The sum loss for KgM-AgNPs is at 82.7%, with ash content of 17.28%. Stage I is attributed to the loss adsorbed moisture by evaporation, stage II is attributed to the breakdown of the polymeric structure of KgM, while stage III, is assigned to the complete carbonization of the materials. From the percent ash, it can be observed that KgM-AgNPs is over nine times more thermally stable than KgM. This agrees with reported work^51^.

With exhaustive instrumental characterizations applied, to reveal the microstructural compositions of KgM and KgM-AgNPs, we proceeded to testing KgM-AgNPs for Hg^2+^ detection in solution. As it is generally sought nowadays, the application of environmentally benign nanomaterials as optical probes to avoid introducing secondary toxic materials, arising from the use of sensing probes, fabricated using hazardous reagents. In this light, we investigated the biocompatibility prospect of KgM-AgNPs, to ascertain its inherent toxicity profile.

### Biocompatibility of KgM-AgNPs

**Figure 6:**
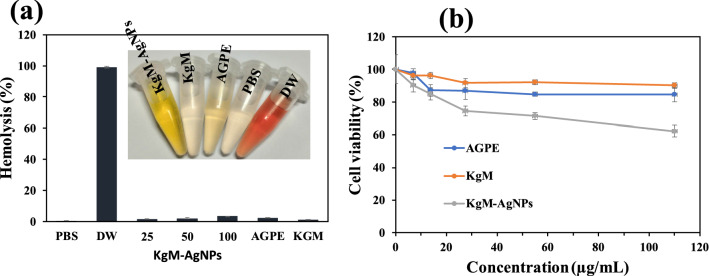
Effect of KgM-AgNPs, AGPE and KgM on (**a**) rat erythrocyte hemolysis and (**b**) viability of mouse fibroblasts.

The hemolytic effect of the nanoparticles as well as the extract and KgM was profiled using rat erythrocytes. RBC hemolysis is one of the simplest approaches for preliminary evaluation of the potential adverse effects of substances in biological systems. Data from the hemolytic assay is presented in Fig. 6a. Following 1 h of co-incubation with fresh erythrocytes at normal physiological temperature, KGM did not display any substantial hemolytic activity at 100 µg/mL. The nanomaterial and extract on the other hand, presented minimal levels of hemolytic activity, i.e., 3.54 and 2.55%, respectively at concentration of 100 µg/mL. Nonetheless, KgM-AgNPs can be non-hemolytic given that it failed to satisfy the specified criteria for hemolytic materials (in vitro hemolytic effect of more than five percent)^52^.

Additionally, the potential cytotoxic effect of KgM-AgNPs was evaluated on mouse L929 fibroblasts. The cells were treated with various concentrations of the nanomaterial as well as the extract and hydrocolloid gum for 24 h. The effect of the samples on viability of mouse fibroblasts is presented in Fig. 6b. It was found that at the highest concentrations tested (110 µg/mL), the extract- and gum-treated cells displayed viabilities of 84.41 and 90.07%, respectively. These values suggested that the extract and gum did not pose any serious adverse effects to the cells. In contrast, KgM-AgNPs displayed a dose-dependent reduction in cell viability, with the least cell viability (62.21%) recorded at the maximum concentration tested (110%). Importantly, it is worth pointing out that at KgM-AgNPs concentration of 55 µg/mL, the fibroblast presented 71.32% viability. This concentration can be regarded as the threshold for the cytocompatibility of KgM-AgNPs, given that it presented a cell viability greater than 70% ^53^. Previous acute toxicity studies of KgM on rats and dogs found that the hydrocolloid was non-toxic. On their part, the European Commission’s EFSA Panel concluded that KgM up to 10 g/kg was of no safety concern to the general population as food additive^54^. Considering that KgM which served as the predominant capping agent in the nanomaterial was innocuous, and the reductant was also non-toxic, it is safe to deduce that the cytocompatible properties of these components contributed to the good safety profile of the KgM-AgNPs. Moreover, while it is safe to conclude that the synthesized probe (KgM-AgNPs) is biocompatible, it´s very important to point out that the probe is found in a different environment of pH and ionic strength during Hg^2+^ detection assay. Thus, the biocompatibility property of KgM-AgNPs may be compromised at the sensing conditions.

### Colorimetric detection of Hg^2+^ based on KgM-AgNPs

AgNPs and AuNPs are popular as optical probes for the detection of diverse analytes in solutions^55,56^. These probes capitalized on the unique localized surface plasmon resonance (LSPR), properties of the nanomaterials. At this condition, colloidal solution of AgNPs and AuNPs display characteristic absorption spectra, unique color and stability. Thus, the addition of particular analyte can upset these properties, while such changes can be correlated to the concentrations of the charged analytes.

With this backdrop, the efficacy of KgM-AgNPs to detect Hg^2+^ in solution was investigated. In order to achieve good sensing capacity, conditions affecting the detection strategy were summarily optimized.

### Optimization of KgM-AgNPs response towards Hg^2+^ detection

The addition of Hg^2+^ to KgM-AgNPs aqueous solution, in PBS solution 50 mM, pH 6.0, in the absence of NaCl do not engender any color change. This may be attributed to the high stability of KgM-AgNPs, arising from the high polymeric spheres shielding the particles from facile interaction in aqueous environment. However, with the addition of NaCl solution, a visible color change could be observed almost instantaneously. This is because NaCl can alter the dielectric surfaces or (surroundings), of the nanoparticles, through the reduction of energy barriers^57^ and thus subject the particles to agglomeration, due to electrostatic forces screening effect from salts^55^.

Having found out that PBS of pH 6.0 is most appropriate for Hg^2+^ detection (Fig. S8), we investigated the effect of different concentrations of NaCl in the reaction mixture of 2 mL final volume. As shown in Fig. S9, the addition of NaCl with final concentrations in the range of 0.005–0.075 M, without Hg^2+^ addition, do not induce immediate color change on the KgM-AgNPs in PBS solution. Moreover, the absorption spectra equally confirmed that NaCl do not educe spectra quenching on KgM-AgNPs after 1 h of addition. To further observe the stability of the colloidal solution (Fig. S9inset), the photo images were collected after 24 h incubation at RT (Fig. S10). As can be seen, NaCl above 0.025 M final concentration (marked with red arrow (Fig. S8), imparted notable color changes. This result shows that KgM-AgNPs under PBS treatment and NaCl can tolerate final NaCl concentration, not beyond 0.025 M. Thus, this concentration was selected for Hg^2+^ detection.

It was also noticed that, under the present detection strategy, Hg^2+^ could impart swift response, consequently, all absorption spectra and photo images were collected after 3 min of RT incubation.

### Sensitivity of KgM-AgNPs as colorimetric probe for Hg^2+^ detection

**Figure 7:**
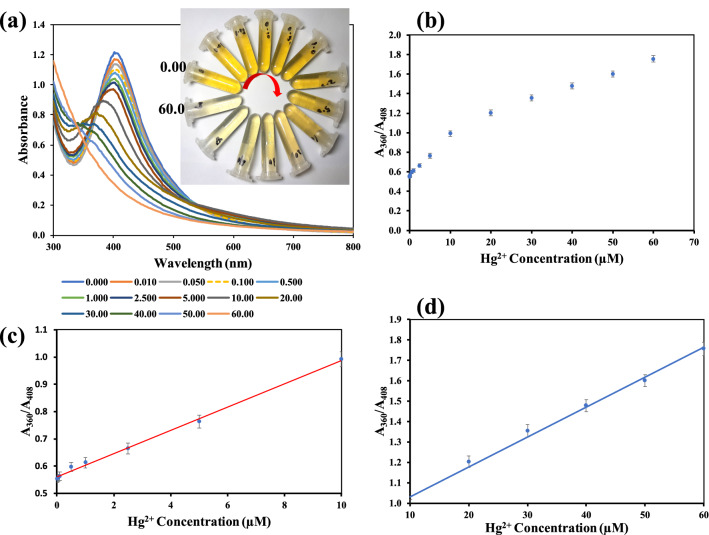
(**a**) UV–vis absorption spectra of KgM-AgNPs under Hg^2+^ charging from 0.00–60.0 µM, inset shows the photo images from 0.00–60.0 µM, in a clockwise direction (**b**) Plot of A_360_/A_408_ vs Hg^2+^ concentration from 0.00–60.0 µM (**c**) Plot of A_360_/A_408_ vs Hg^2+^ showing linearity within 0.010–10.0 µM Hg^2+^ (**d**) Plot of A_360_/A_408_ vs Hg^2+^ showing linearity within 10.0–60.0 µM of Hg^2+^.

Sensitivity is a measure of the change in analyte response to concentration. Hence different concentrations of Hg^2+^ (0.00–60.0 µM) were charged on KgM-AgNPs in PBS 50 mM, pH 6.0, and NaCl concentration of 0.025 M final concentration. As shown in Fig. 7a, the addition of Hg^2+^, results in progressive decrease in absorption spectra of KgM-AgNPs, with a blue shift. The color of KgM-AgNPs transited from sparkling yellow to colorless (Fig. 7a inset). The plot of absorbance ratios (A_360_/A_408_), of colorless KgM-AgNPs (A_360_), to yellow color KgM-AgNPs (A_408_), against Hg^2+^ concentrations, over the full range 0.00–60.0 µM, is shown in Fig. 7b. Accordingly, the ratio increases as the concentration of Hg^2+^ increases, confirming that the color of KgM-AgNPs fades away as Hg^2+^ ion concentration increases in the reaction mixture. The plot can be fitted into two linear ranges, as depicted in Fig. 7c,d. The calibration plots for the two Hg^2+^ concentrations ranges include: (A_360_/A_408_) = 0.0427 [Hg^2+^] + 0.5604, R^2^ = 0.9964, for Hg^2+^ concentration 0.010–10.0 µM (Fig. 7c) and (A_360_/A_408_) = 0.0147 [Hg^2+^] + 0.8841, R^2^ = 0.9909, for Hg^2+^ concentration 10.0–60.0 µM (Fig. 7d). The limit of detection (LOD) and the limit of quantification (LOQ) were estimated using the equation, 3.3 S_(y/x)_ / slope and 10 S_(y/x)_ / slope, respectively. Units explained as follows: S_(y/x)_ is the standard deviation calculated from the regression, while slope is from the calibration plot^58^, from Fig. 7c. The LOD and LOQ were calculated to be 3.25 and 9.85 nM. The obtained LOD value is better than or fairly comparable to some of the reported works for Hg^2+^ detection in solution (Table 1). As can be observed, the LOD of this work would compare fairly with other reported works. Though, there are similar works for Hg^2+^ detection based on Hg^2+^ mediated degradation of AgNPs^19,20^, the use of highly toxic chemicals such as sodium borohydride for AgNPs preparation^15^, would be less desirous under the “green chemistry” discourse. It must be equally stressed that the use of *A. gangetica* in this work*,* a notorious garden weed, with extensive characterization of its physico-chemical properties, presents an interesting information into the body of knowledge. It can also be argued that the plant has been successfully valorized, with its use as an effective reductant in the present synthesis of KgM-AgNPs.

**Table 1:** Comparison of KgM-AgNPs sensor with reported works towards Hg^2+^ detection in solution.

Detection method	Linear range (µM)	Limit of detection (LOD) (nM)	References
Electrochemistry (Cyclic voltammetry)	0.001–0.50	0.014	23
Electrochemistry (Differential pulse voltammetry)	0.001–0.20	0.38	24
Colorimetry (Au@DTSP/CRN)	0.10–0.35 & 0.35–4.00	28.5	17
Colorimetry(Au@S-g-C_3_N_4_)	0.10–0.50	0.275	18
Colorimetry(Gum Kondagogu-AgNPs)	0.050–0.90	50.0	19
Sodium alginate-AgNPs	0.025–60.0	5.29	20
Fluorescence(Nitrogen doped carbon qdots)	0.0–5.0	17.0	21
Fluorescence	0.0–5.0	10.5	22
Fluorescence (carbon dots)	0.0–5.8	7.6	61
Colorimetry(KgM-AgNPs)	0.010–10.0 and 10.0–60.0	3.25	This work

Au@DTSP/CRN = creatinine on 3, 3′-dithiodipropionic acid di (N-hydroxysuccinimide ester) functionalized gold nanoparticles; Au@S-g-C_3_N_4_ = gold nanoparticles assembled on Sulphur doped graphitic carbon; qdots = quantum dots.

Moreover, the precision of the detection strategy was estimated and expressed in terms of relative standard deviation (RSD%). Here, the absorption spectra of two Hg^2+^ concentrations at 5.0 and 30.0 µM were monitored (n = 10), for same day (intra-day precision) and for three consecutive days (inter-day precision). The RSD were estimated as 1.8% and 2.5%, for intra-day and inter-day precision respectively. This confirmed that the KgM-AgNPs can reliably detect Hg^2+^ in solution without much data variability, hence good reproducibility.

**Figure 8:**
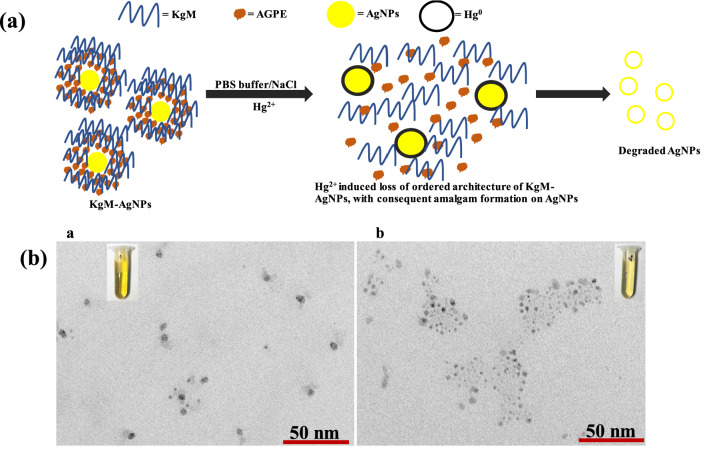
(**a**) Representation of the mechanistic basis of KgM-AgNPs towards the detection of Hg^2+^ in aqueous solution (**b**) TEM images of KgM-AgNPs under Hg^2+^ treatment at 0.00 and 30.0 µM, inset shows the photo images of the different treatment.

Furthermore, the mechanistic explanation on KgM-AgNPs as optical probe for Hg^2+^ in solution is shown in Fig. 8. We have explained through the synthesis steps of KgM-AgNPs that AGPE facilitated the reduction of Ag^+^, while KgM polymer sphere provided further stabilization to the formed AgNPs. Thus, Fig. 8a depicted our KgM-AgNPs with excess AGPE and KgM polymer spheres, held in place through hydrogen bonding interaction. However, with the addition of PBS, NaCl and Hg^2+^ in solution, the well-ordered architecture between AGPE and KgM, holding AgNPs in shape is disrupted. Consequently, Hg^2+^ could have direct access to Ag^0^ and thus a redox reaction is set in place between Hg^2+^ and Ag^0^. The standard electrode potential of Ag^+^/Ag^0^ is 0.80 V, while that of Hg^2+^ /Hg^0^ is 0.85 V^59^. Accordingly, Hg^2+^ would serve as an oxidizing agent, in a reaction involving Ag^0^ and Hg^2+^ in solution. More, Hg^2+^ will be reduced, while Ag^0^ is oxidized to Ag^+^. As seen in Fig. 8a, Hg^2+^ is reduced to Hg^0^ and it was deposited on the surface of Ag^0^ to form [Ag-Hg] complex or amalgam. This amalgam is responsible for the gradual reduction in the absorption spectra of KgM-AgNPs, with a blue shift. The sparkling yellow color of KgM-AgNPs are completely lost at a high Hg^2+^ concentration due to the etching or fading away of AgNPs, with concomitant colorless solution development. We further substantiated the proposed mechanism with the acquisition of TEM images after Hg^2+^ treatment on KgM-AgNPs at 0.00 and 30.0 µM (Fig. 8b). As revealed in Fig. 8b a), the blank solution i.e. KgM-AgNPs with 0.00 µM Hg^2+^, the particles are still well dispersed in solution, while the sparkling yellow color of KgM-AgNPs are sharply observed (Fig. 8b a-inset). However, with the injection of Hg^2+^ of 30.0 µM, the particles are clearly observed to be degraded (Fig. 8b b) and the faint yellow color of KgM-AgNPs are depicted in Fig. 8b b-inset. These results lay more credence to the proposed mechanism in confirmation of Hg^2+^ mediated oxidation and further degradation of KgM-AgNPs in solution.

### Selectivity

**Figure 9:**
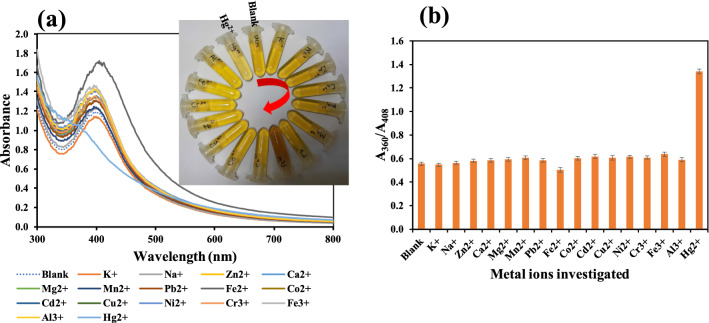
(**a**) UV–vis absorption spectra of KgM-AgNPs under charging with different metal ions, from blank-Hg^2+^ (**b**) Plot of A_360_/A_408_ vs various metal ions of environmental significance, Hg^2+^ concentration was at 30.0 µM, while other metal ions were fixed 150.0 µM.

The selectivity of KgM-AgNPs probe was estimated by charging different commonly existing metal ions in environmental samples on the developed colorimetric probe. The concentration of Hg^2+^ was maintained at 30.0 µM, while other common metal ions were at 150.0 µM. As shown in Fig. 9a, only the addition of Hg^2+^ resulted in blue shift in absorption spectra (Fig. 9a), while the color fading, was observed only in the presence of Hg^2+^ (Fig. 9a(inset)), even when other metal ions are at five-fold concentration higher then Hg^2+^. Further the absorbance ratio, A_360_/A_408_ against the tested metal ions (Fig. 9b), revealed that only Hg^2+^ educed a significant increment, in comparison with the blank. However, Fe^2+^ formed a deep brown color with an absorption spectra enhancement (Fig. 9a), which may be attributed to color development of ferrous salts. This same observation has been reported^60^. This shows the KgM-AgNPs probe is selective towards only Hg^2+^ and thus can be applied to its detection even in the presence of other metal ions. Additional interference study was investigated by mixing the tested ions with Hg^2+^, after which the absorption spectra were collected. The absorbance ratio (A_360_/A_408_) was compared with the value obtained for Hg^2+^ only, as shown in Fig. S11. There existed no significant differences (P ≥ 0.05), between Hg^2+^ response (alone) and Hg^2+^ mixed with other metal ions, which authenticated the capacity of KgM-AgNPs to detect Hg^2+^ in complex environmental.

### Real sample application

The practical application of KgM-AgNPs towards Hg^2+^ detection in real sample was tested on reservoir water from the Prince of Songkhla University, Hatyai, campus. The water sample was briefly subjected to centrifugation and passed through 0.22-micron membrane filter. Afterwards, some part of the water was submitted for Hg^2+^ quantification using ICP-OES. Hg^2+^ was not detected in the sample and thus, the standard addition method was applied, where different concentrations of Hg^2+^ were spiked into the collected water sample. The Hg^2+^ spiked sample was run on KgM-AgNPs as proposed in section on analytical detection of Hg^2+^ in solution at the materials and method. The obtained absorption spectra were converted to concentration using the standard calibration plots obtained. The recovery was estimated using the recovery equation in Table 2. As estimated, the recovery values ranged between 95.1 and 98.7%, with RSD less than 5.0%. This shows the present assay possess reliable accuracy for Hg^2+^ determination in environmental sample.

**Table 2:** Detection of Hg^2+^ in water sample using KgM-AgNPs probe.

Added concentration (µM)	Found concentration (µM)	Recovery (%)	RSD % (n = 3)
Unspiked (0.00)	0.00	–	–
0.050	0.0487 ± 0.08	97.4	1.6
1.00	0.987 ± 0.12	98.7	2.3
5.00	4.753 ± 0.15	95.1	3.2

Recovery = (C_s_ − C_us_)/C_st_ $\times$ 100%, C_s_ is the concentration of spiked sample, C_us_ is the concentration of unspiked sample, C_st_ is the concentration of standard added.

## Conclusion

In this contribution, we have reported the synthesis of biocompatible Konjac glucomannan (KgM) stabilized AgNPs using *Asystasia gangetica* phenolic extract (AGPE) as the reducing agent, towards the fabrication of highly stable, dispersed KgM-AgNPs. This work revealed for the first time the use of the plant in AgNPs synthesis, while also delineating the very first synthesis of KgM-stabilized AgNPs at room temperature. The bioactive compounds identified in the plant holds great promise on further investigation of A. gangetica for pharmaco-biological applications. The synthesized KgM-AgNPs showed average particles distribution between 10 and 15 nm, hydrodynamic diameter of 60.2 ± 1.5 nm and zeta potential of −28.8 ± 2.1 mV. The biocompatibility of KgM-AgNPs was demonstrated on mouse L929 fibrobroblast and rat red blood cell, which validated that the synthesized KgM-AgNPs is non-toxic to the tested cells. The addition of Hg^2+^ to KgM-AgNPs in solution, with PBS pH 6.0, 50 mM and NaCl (0.025 M final concentration), sparkling yellow color solution of KgM-AgNPs was progressively faded to colorless, within 3 min, with concomitant blue shift in absorption spectra. Absorbance ratio, A_360_/A_408_ was found to be linear with Hg^2+^ concentrations over two ranges 0.010–10.0 & 10.0–60.0 µM. The estimated LOD was 3.25 nM. The practical effectivity of the developed probe was demonstrated, with Hg^2+^ spiked-recovery estimation in real water sample, with satisfactory accuracy. Unlike some reported detection strategy for Hg^2+^ where toxic materials have been used for the nanomaterial’s fabrication, this work is completely green in view of the contributing materials adopted. Thus, this work showcased a practical approach to the synthesis of highly sensitive and stable plasmonic AgNPs with reliable detection strength.

## Supplementary Information

Supplementary Information.

## Data Availability

The datasets generated and/or analyzed during the current study are not publicly available due to confidentiality concerns but are available from the corresponding author on reasonable request.

## Abbreviations

AgNPs: Silver nanoparticles
KgM: Konjac glucomannan
KgM-AgNPs: AGPE based Konjac glucomannan stabilized silver nanoparticles
AGPE: *Asystasia gangetica* Phenolic extract
RT: Room temperature
