# Supplementary material for: Synthesis of biocompatible Konjac glucomannan stabilized silver nanoparticles, with Asystasia gangetica phenolic extract for colorimetric detection of mercury (II) ion
Source: Sci Rep. 2022 Jun 2;12:9176. doi: 10.1038/s41598-022-13384-x (PMC9163164; doi:10.1038/s41598-022-13384-x)
Supplement: Supplementary file 1 — Supplementary Information. [file 41598_2022_13384_MOESM1_ESM.docx]

**Supplementary Materials**

**Synthesis of biocompatible Konjac glucomannan stabilized silver nanoparticles, with *Asystasia gangetica* phenolic extract for colorimetric detection of mercury (II) ion**

**Titilope John Jayeoye^1*^, Fredrick Nwude Eze^2,3**^, Opeyemi Joshua Olatunji^4^ & Andrew Aondoaver Tyopine^1^**

^1^ Department of Chemistry, Faculty of physical sciences, Alex-Ekwueme Federal University, Ndufu-Alike Ikwo, Abakaliki, Ebonyi State, Nigeria

^2^ Faculty of Pharmaceutical Sciences, Prince of Songkhla University, Hat Yai, Songkhla 90112, Thailand

^3^ Drug Delivery System Excellence Center, Prince of Songkhla University, Hat Yai, Songkhla 90112, Thailand

^4^ Traditional Thai Medical Research and Innovation center, Faculty of Traditional Thai Medicine, Prince of Songkhla University, Hat Yai, 90110, Thailand

**Corresponding authors:**

* Department of Chemistry, Faculty of Physical Sciences, Alex-Ekwueme Federal University, Ndufu-Alike Ikwo, P.M.B. 1010, Abakaliki, Ebonyi State, Nigeria

Email address: [titilope12@gmail.com](mailto:titilope12@gmail.com) **(T.J. Jayeoye)**

**Faculty of Pharmaceutical Sciences, Prince of Songkhla University, Hat Yai, Songkhla 90112, Thailand

Email address: fredrick.e@psu.ac.th **(F.N. Eze**

|  |  |  |  |  |  |  |  |  |
| --- | --- | --- | --- | --- | --- | --- | --- | --- |
| S/N | **Name (Tentative ID)** | **Formula** | **m/z** | **Accurate mass (Da)** | **RT (min)** | **Score (DB)** | **Mass DB (calculate)** | **Diff**  **(ppm)** |
| 1 | Kelampayoside A | C_20_H_30_O_13_ | 523.1677 | 478.1695 | 4.932 | 97.67 | 478.1686 | -1.82 |
| 2 | Caryoptosidic acid | C_16_H_24_O_11_ | 391.1241 | 392.1313 | 5.033 | 82.46 | 392.1319 | 1.31 |
| 3 | Pantothenic Acid | C_9_H_17_NO_5_ | 218.1035 | 219.1108 | 5.635 | 99.38 | 219.1107 | -0.55 |
| 4 | Bufotenine O-glucoside | C_18_H_26_N_2_O_6_ | 411.1776 | 366.1794 | 5.66 | 98.87 | 366.1791 | -0.74 |
| 5 | Verbasoside | C_20_H_30_O_12_ | 461.1662 | 462.1735 | 5.886 | 98.49 | 462.1737 | 0.45 |
| 6 | Methyl salicylate O-[rhamnosyl-(1->6)-glucoside] | C_20_H_28_O_12_ | 459.1518 | 460.1591 | 6.564 | 95.96 | 460.1581 | -2.14 |
| 7 | trans-p-Coumaric acid 4-glucoside | C_15_H_18_O_8_ | 325.0938 | 326.1011 | 6.615 | 96.56 | 326.1002 | -2.82 |
| 8 | (1x,2x)-Guaiacylglycerol 3-glucoside | C_16_H_24_O_10_ | 375.1305 | 376.1378 | 6.765 | 97.69 | 376.1369 | -2.16 |
| 9 | 2-[4-(3-Hydroxypropyl)-2-methoxyphenoxy]-1,3-propanediol 1-xyloside | C_18_H_28_O_9_ | 387.1659 | 388.1731 | 6.878 | 98.96 | 388.1733 | 0.47 |
| 10 | Magnoloside B | C_35_H_46_O_20_ | 831.2558 | 786.2573 | 6.966 | 97.5 | 786.2582 | 1.18 |
| 11 | Quercetin 3-[p-coumaroyl-(->6)-glucosyl-(1->2)-glucosyl-(1->2)-glucoside] | C_42_H_46_O_24_ | 933.2325 | 934.2396 | 7.142 | 95.85 | 934.2379 | -1.83 |
| 12 | 19-Hydroxycinnzeylanol 19-glucoside | C_26_H_42_O_13_ | 561.2544 | 562.2615 | 7.945 | 96.18 | 562.2625 | 1.85 |
| 13 | Glucoliquiritin apioside | C_32_H_40_O_18_ | 711.2152 | 712.2226 | 8.046 | 96.42 | 712.2215 | -1.64 |
| 14 | Isoacteoside | C_29_H_36_O_15_ | 623.1994 | 624.2065 | 8.096 | 97.19 | 624.2054 | -1.78 |
| 15 | 1-Octen-3-yl primeveroside | C_19_H_34_O_10_ | 467.2139 | 422.2157 | 8.887 | 98.18 | 422.2152 | -1.08 |
| 16 | 8-Acetoxypinoresinol 4-glucoside | C_28_H_34_O_13_ | 577.193 | 578.2 | 9.025 | 98.43 | 578.1999 | -0.17 |
| 17 | Glaucarubinone | C_25_H_34_O_10_ | 493.2081 | 494.2155 | 9.978 | 95.31 | 494.2152 | -0.62 |

Rt: retention time

**Table S1:** Chemical profile of bioactive compounds present in *Asystasia gangetica* extract obtained by UPLC-ESI-QTOF-MS analysis


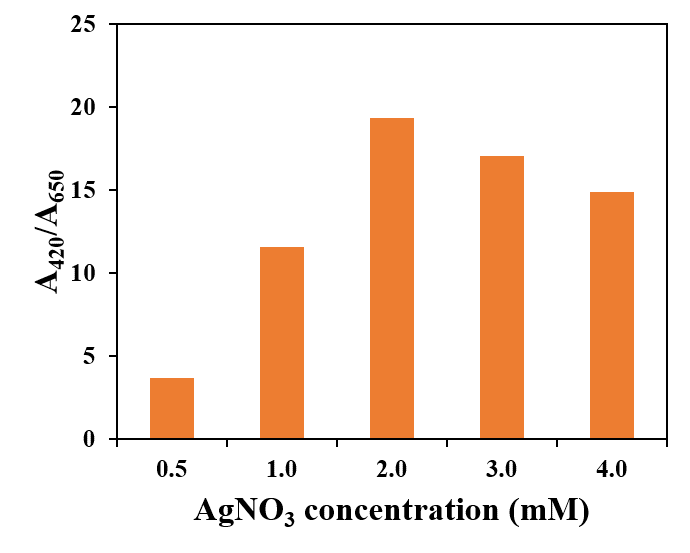


**Figure S1.** Plot of absorbance ratios (A_420_/A_650_) Vs AgNO_3_ concentrations, to obtain information on the nanoparticle’s dispersity and stability.


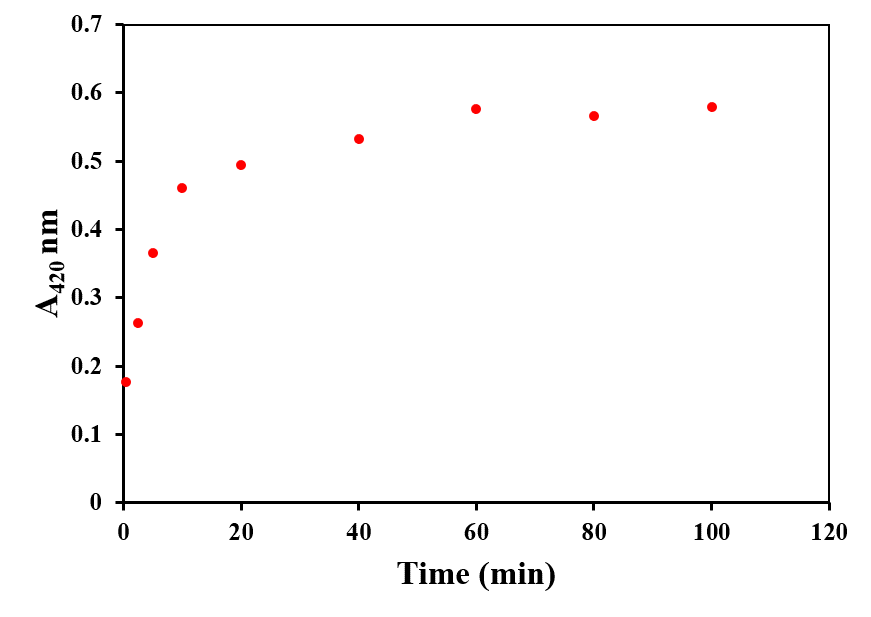


**Figure S2.** Plot of A_420_ nm against Time (min) of KgM-AgNPs synthesized at optimal condition.


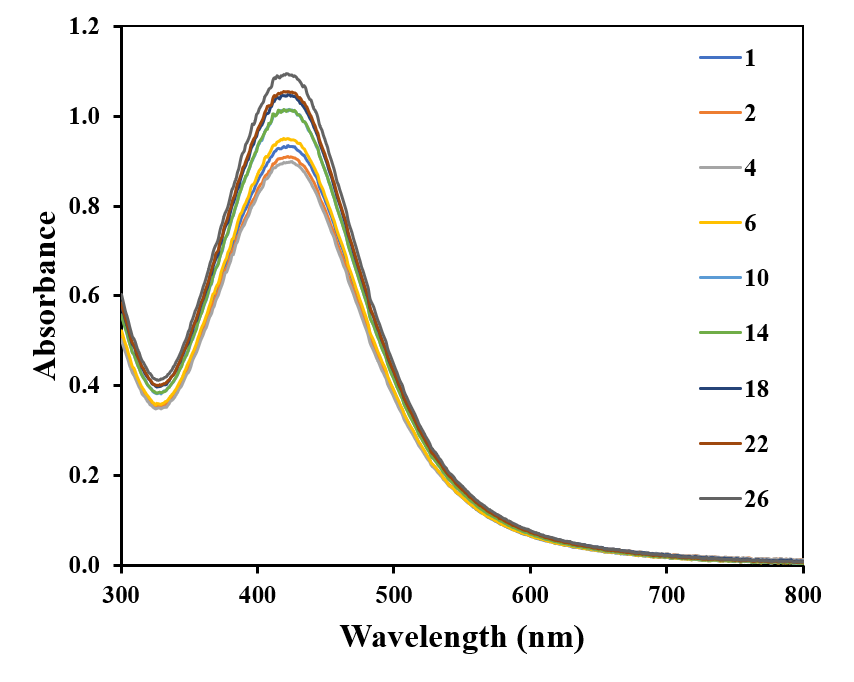


**Figure S3**. UV-vis absorption spectra of KgM-AgNPs monitored for 26 weeks.

**D_h_ = 60.2 ± 1.5 nm**

**Figure S4**. Hydrodynamic diameter of KgM-AgNPs from DLS.

**Figure S5.** Plot of pH against Zeta potential of KgM-AgNPs, revealing the pH dependence of KgM-AgNPs

**Figure S6**. EDS patterns of A. KgM and B. KgM-AgNPs

**Figure S7.** Elemental mapping of A. KgM and B. KgM-AgNPs.

| **Sample** | **Stages** | **Onset Temperature (°C)** | **Temperature ranges (°C)** | **IDT (°C)** | **IPDT (°C)** | **Mass loss (%)** | **Ash (%)** |
| --- | --- | --- | --- | --- | --- | --- | --- |
| KgM | I | 51.44 | 51.44-259.84 | 51.44 | 999.30 | Y = 4.53 + 4.28 | **2.03** |
|  | II | 259.84 | 259.84-386.18 |  |  | Y = 74.76 +4.52 |  |
|  | III | 386.18 | 386.18-999.30 |  |  | Y = 5.83 +4.05 |  |
|  |  |  |  |  |  | **Total = 97.97** |  |
| KgM-AgNPs | I | 51.48 | 51.48-237.74 | 51.48 | 999.30 | Y = 1.72 +7.50 + 19.94 | **17.28** |
|  | II | 237.74 | 237.74-341.54 |  |  | Y = 24.31 +4.62 |  |
|  | III | 341.54 | 341.54-999.30 |  |  | Y = 21.55 +3.08 |  |
|  |  |  |  |  |  | **Total =** **82.72** |  |

**IDT: Initial decomposition temperature; IPDT: Integral procedural decomposition temperature.**

**Table S2.** TGA of KgM and KgM-AgNPs

**Figure S8.** Plot of buffer pH against absorbance ratio A_360_/A_408_, under Hg^2+^ concentration at 30.0 µM.


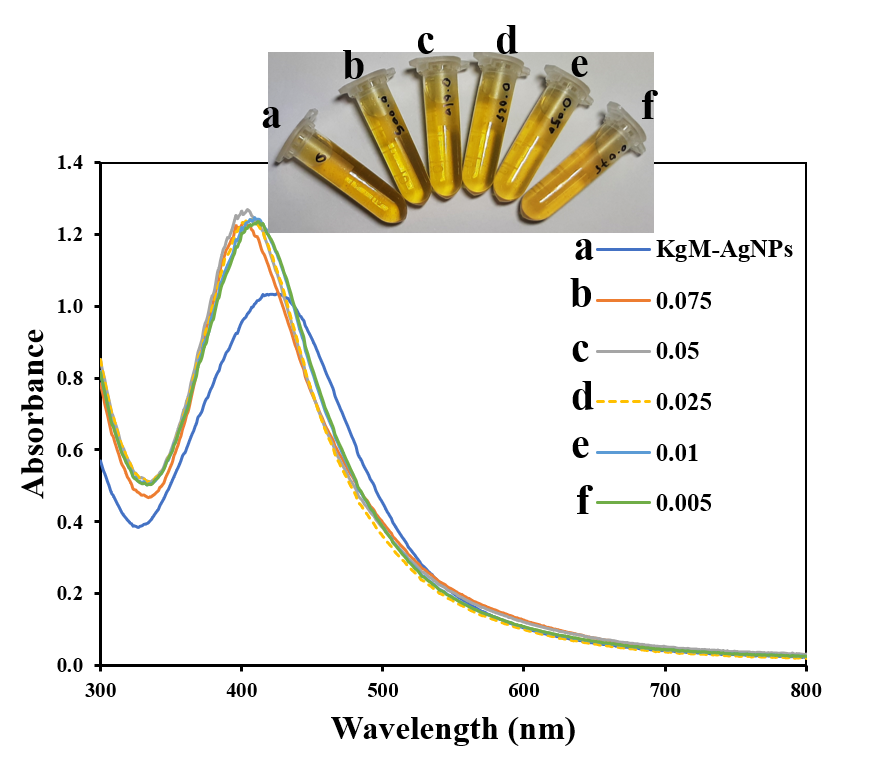


**Figure S9.** UV-vis absorption spectra of KgM-AgNPs, in PBS 50 mM, pH 6.0, under different concentrations of NaCl, a 0.00 (KgM-AgNPs) b 0.075 c 0.050 d 0.025 e 0.010 and f 0.005M, final concentration. The photo images and UV-vis spectra were collected after 1hr.


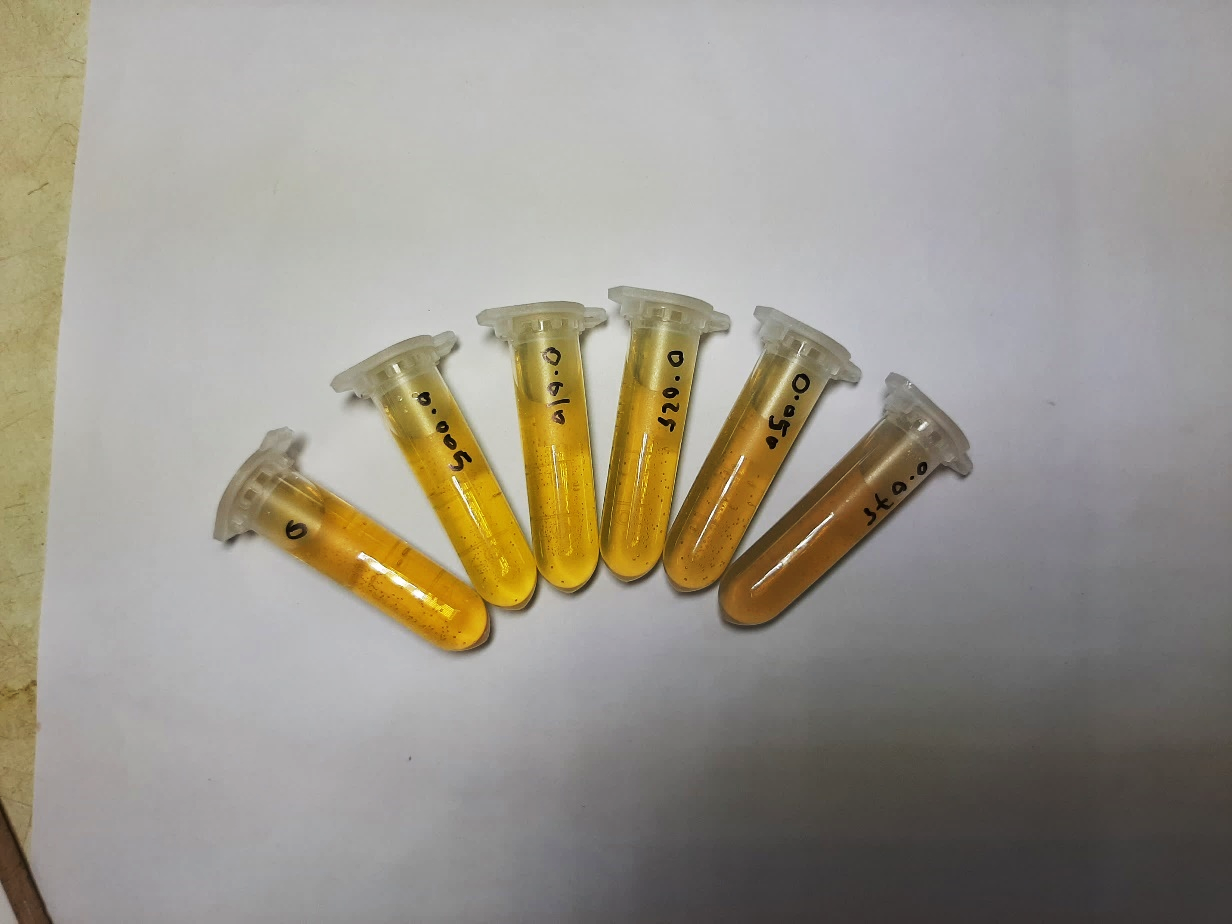


**Figure S10.** Photo images of Fig.S6 colloidal solution after 24 hr incubation at RT.


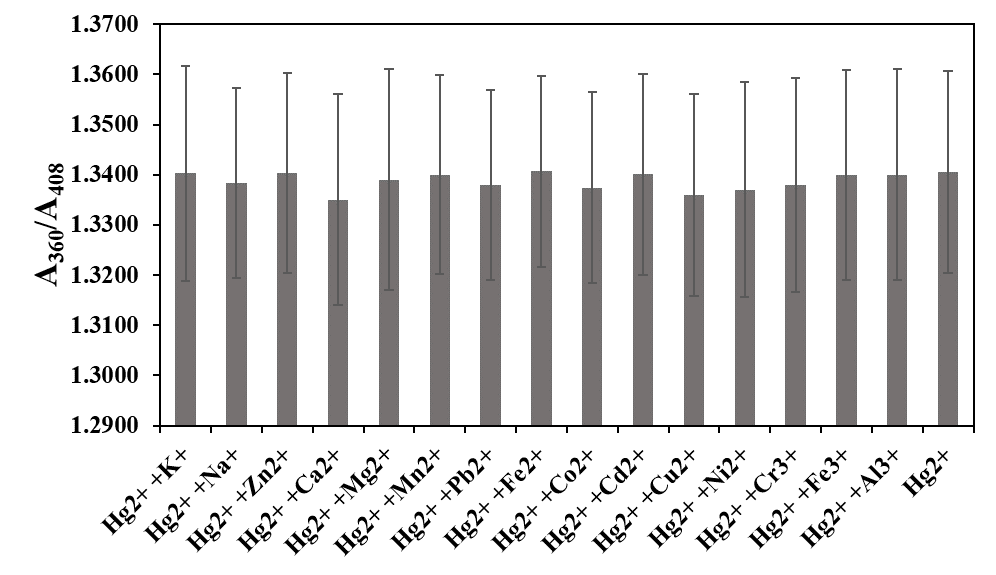


**Figure S11.** Plot of absorbance ratio (A_360_/A_408_) against Hg^2+^ mixed with various metal ions. Hg^2+^ concentration was at 30.0 µM, other metal ions were at 150.0 µM.
